# The influence of the rib cage on the static and dynamic stability responses of the scoliotic spine

**DOI:** 10.1038/s41598-020-73881-9

**Published:** 2020-10-09

**Authors:** Shaowei Jia, Liying Lin, Hufei Yang, Jie Fan, Shunxin Zhang, Li Han

**Affiliations:** 1 Key Laboratory for Biomechanics and Mechanobiology of Ministry of Education, Beijing Advanced Innovation Center for Biomedical Engineering, School of Biological Science and Medical Engineering, Beihang University, Beijing, China; 2 School of Mechanical Engineering, Hebei University of Technology, Tianjin, China; 3 School of Medical Imaging, Tianjin Medical University, Tianjin, China

**Keywords:** Biomedical engineering, 3-D reconstruction

## Abstract

The thoracic cage plays an important role in maintaining the stability of the thoracolumbar spine. In this study, the influence of a rib cage on static and dynamic responses in normal and scoliotic spines was investigated. Four spinal finite element (FE) models (T1–S), representing a normal spine with rib cage (N1), normal spine without rib cage (N2), a scoliotic spine with rib cage (S1) and a scoliotic spine without rib cage (S2), were established based on computed tomography (CT) images, and static, modal, and steady-state analyses were conducted. In S2, the Von Mises stress (VMS) was clearly decreased compared to S1 for four bending loadings. N2 and N1 showed a similar VMS to each other, and there was a significant increase in axial compression in N2 and S2 compared to N1 and S1, respectively. The U magnitude values of N2 and S2 were higher than in N1 and S1 for five loadings, respectively. The resonant frequencies of N2 and S2 were lower than those in N1 and S1, respectively. In steady-state analysis, maximum amplitudes of vibration for N2 and S2 were significantly larger than N1 and S1, respectively. This study has revealed that the rib cage improves spinal stability in vibrating environments and contributes to stability in scoliotic spines under static and dynamic loadings.

## Introduction

Scoliosis, a three-dimensional deformity, prevents healthy development. It results in body deformation, affects growth and development in adolescents, and quality of life in adults. When scoliosis patients are exposed to a moving environment, the asymmetry of the distribution of the load on each lateral convex segment may result in them experiencing higher stresses and strains, exacerbating deformation and leading to more serious damage^1,2^. A number of studies have reported that subjects with scoliosis exhibit a higher risk of lower back pain (LBP) than healthy individuals^3,4^. Long-term whole-body-vibration (WBV) contributes to LBP and aggravates the deformation already experienced in scoliosis patients^5^. The stress and strain generated in a scoliotic spine subjected to a dynamic load are two to threefold greater than a static load of the same magnitude^6,7^.

Many researchers have studied the structural characteristics, tendency to strain, and stress distribution of normal and scoliotic lumbar, and scoliotic thoracic spines without the rib cage when subjected to external static (or dynamic) loads^8^, but they have generally neglected the role of the rib cage (the cage structure comprising the ribs, sternum and costal cartilage) when investigating spinal stability. There are a few studies which have examined the mechanical impact of the rib cage on thoracic stability^9,10^. Watkins^11^ and Mannen^12^ used cadaveric specimens to study the effect of the rib cage on stability and stiffness of a normal spine exposed to static loading. Because the dynamic characteristics of normal and scoliotic spines are critical for understanding the effects of WBV on spines and their stability, the responses of the rib cage to the stability of scoliotic and normal spines also need to be examined.

Finite element analysis (FEA) is an effective method for quantifying the biomechanical characteristics of human organs, and the spine in particular. The biomechanical characteristics of lumbar facet joints were studied using the FEA method^13^. Since many researchers have established FE models to simulate spinal biomechanical characteristics and predict potential clinical risks, a spinal FE model was established to investigate the dynamic responses of scoliotic spines under axial cyclic loads^4^. A previous study which investigated the vibration responses of three kinds of scoliotic lumbar spine using the FEA method^14^ showed that vibration was strongly harmful to the lumbar spine, especially to scoliosis segments. These studies have indicated that FEA methods can be used to evaluate the biomechanical responses of the spine, including static and dynamic responses under different loadings. However, vibration characteristics and the effects of the rib cage on stability are still not clear in either normal or scoliotic spines. Therefore, the aim of this study was to comprehensively study the vibration characteristics and the effects of the rib cage on the stability in normal and scoliotic spines under static and dynamic WBV loadings.

In this study, spinal FE models, including a normal spine with rib cage (N1), a normal spine without rib cage (N2), a scoliotic spine with rib cage (S1) and a scoliotic spine without rib cage (S2) were established based on CT images. Static analysis of the four FE models was performed to observe the distribution of stress and strain on spines. Modal analysis of the four FE models was conducted, and the resonant frequencies and modes of vibration around 3–8 Hz were obtained. Steady-state analysis of the four FE models was performed to compare the differences in dynamic WBV characteristics of the normal and scoliotic FE models with and without a rib cage, and to study the effects of the rib cage on the dynamic stability of the scoliotic spine. These results will help to raise awareness of the importance of the rib cage in stabilization in clinical practice, and will help scoliosis patients to understand their spine’s characteristics and prevent further deterioration.

## Materials and methods

### Data source

A typical Lenke 1BN scoliosis subject (male, 25 years old, 71.2 kg, 165 cm, BMI = 26.1 kg/cm^2^, Cobb angle: 67° for thoracic spine and 46° for lumbar spine; no other musculoskeletal diseases) and a healthy subject (male, 27 years old, 69.5 kg, 173 cm, BMI = 23.2 kg/cm^2^, no musculoskeletal diseases) were recruited for this study. Written informed consent was obtained from both participants and research was approved by the Ethics Commission of Tianjin Medical University with an ethical report. All methods used in this study were performed in accordance with the relevant guidelines and regulations in the Declaration of Helsinki. A 64-slice spiral CT (Siemens, Germany) was used to scan the scoliosis patient and the healthy volunteer from the upper portion of T1 to the end of the sacrum in Beijing Union Hospital. The scanning parameters were as follows: 120 kV, 211.20 mAs, 0.625 mm scanning thickness, 0.75 mm × 0.75 mm in-plane resolution and 512 × 512 matrix. A total of 867 images in DICOM format were obtained for each scan. Authorization was obtained to use this data.

### Establishment of spinal FE models

**Figure 1:**
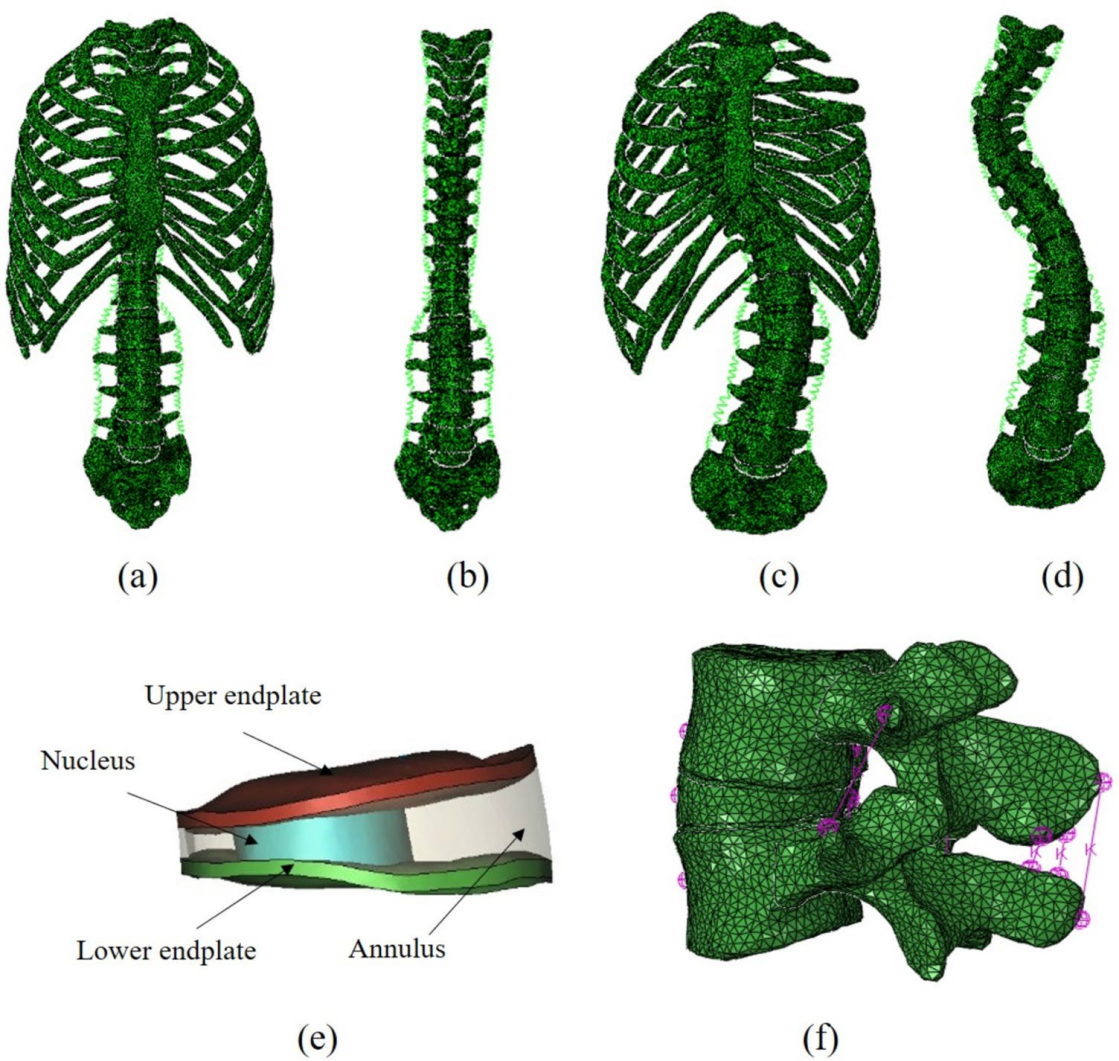
The four FE models and their details. (**a**) Normal spine with rib cage (N1); (**b**) Normal spine without rib cage (N2); (**c**) Scoliotic spine with rib cage (S1); (**d**) Scoliotic spine without rib cage (S2); (**e**) The detail disc; (**f**) The segment of L4-l5 and ligaments.

Three-dimensional models of both parts of the vertebrae were established using Mimics 16.0 software (Materialize Inc., Belgium, https://www.materialise.com/en/medical/mimics-innovation-suite/mimics) based on CT images. Boolean calculation was performed to divide the smoothed model of the vertebrae into the posterior part and cortical bone with a 1 mm thickness. These intervertebral discs include an annulus, nucleus pulposus (which takes up one-third of the discs and is located at the posterior end), and upper and lower endplates which were 1 mm thick. A detailed disc is shown in Fig. 1. A thoracic structure was created with a sternum, ribs, costal cartilage, and rib joints (although limited by CT data sharpness, rib transverse joints were omitted).

In this study, all the parts were meshed in Mimics, each edge of the element size was set to about 1 mm, and all vertebrae and discs were C3D4 elements. The material properties were homogeneous and isotropic, as according to published literature^14–17^. The types and material properties of the finite element models are shown in Table 1. The principal ligaments connecting the thoracic vertebrae include the supraspinous ligament (SSL) in the thoracolumbar segment, interspinous ligament (ISL), anterior longitudinal ligament (ALL), posterior longitudinal ligament (PLL), intertransverse ligament (ITL) and ligamentum flavum (LF). In this study, linear tension springs were used to simulate ligaments. The definition of stiffness (Spring stiffness = Elastic modulus × Section-area/Mean length) and the material properties of the ligaments were based on accepted data in the published literature^18^. The N1, N2, S1, and S2 FE models are shown in Fig. 1.

**Table 1:** Element type and material parameters of the FE models.

Structure	Elastic modulus (MPa)	Poisson ratio	Section-area (mm^2^)	Mean length (mm)	References
Cortical bone	12,000	0.30			^14^
Cancellous bone	150	0.30			^14^
Posterior body	3500	0.30			^14^
End plate	100	0.40			^15^
Annulus ground substance	4.2	0.45			^17^
Nucleus pulposus	1	0.499			^17^
Rib cage	12,000	0.30			^16^
Intercostal cartilage	300	0.40			^16^
Sternal	12,000	0.30			^16^
ALL	7.8		22.4	20	^18^
PLL	10		7.0	12	^18^
SSL	8		10.5	22	^18^
ISL	10		14.1	13	^18^
ITL	10		0.6	32	^18^
LF	17		14.1	15	^18^

### Boundary and loading conditions

Tie constraints which prevented sliding displacement were established between the all adjacent parts in the FE models of spines with and without rib cages. In addition, all freedom degrees of the two sides near the sacroiliac plane of the sacrum were set entirely constrained according to the anatomical characteristics of the spinal structure. Loadings on the FE models differed according to the conditions shown below. The four FE spinal models were established using Abaqus 6.14 (Dassault SIMULIA Inc., France, https://www.3ds.com/products-services/simulia/products/abaqus/) finite element analysis software, as shown in Fig. 1.

For static analysis, given the complexity of human body movement during daily life and travel, spine movements were simplified to postures such as left and right lateral bending, flexion, extension, and axial compression. According to the mass of a head, a 100 N force was added from the posterior to the anterior on T1 for flexion, from the anterior to the posterior on T1 for extension, from right to the left on T1 for left lateral bending, from the left to the right on T1 for right lateral bending, and applied from the top to the bottom on T1 for vertical compression^10^. Static analysis of daily postures of the spinal structure can reflect the static characteristics of the complicated motion of the human body. Static characteristics of the four models were compared for each posture.

For dynamic analysis, the resonant frequencies and modes of vibration were obtained from the modal analysis of N1, N2, S1 and S2. Consequently, the dynamic responses of the four FE models to external excitation were further analyzed. In modal analysis, a point mass of 10.5 kg was added to the upper surface of T1 to simulate the effects of the mass of the upper body such as the head and neck on FE models^19,20^. Modal analysis of the four FE models could only reflect the intrinsic properties of the FE models, but steady-state analysis of the FE models could be used to obtain spinal response to a real vibrational environment. In steady-state analysis, in addition to a point mass of 10.5 kg, a sinusoidal excitation force of ± 40 N was added to the upper surface of T1 to simulate forced vibration of the body in the axial direction^4^, which was thought to be related to human body vibration during vehicular transport^21^. The damping coefficient of the spine was 0.08^22^. All degrees of freedom on both sides of the sacrum were completely fixed in all analyses^23,24^. Steady-state analysis was used to obtain the effects of the rib cage on the structural stability of the thoracolumbar spine.

### FE model validation

**Figure 2:**
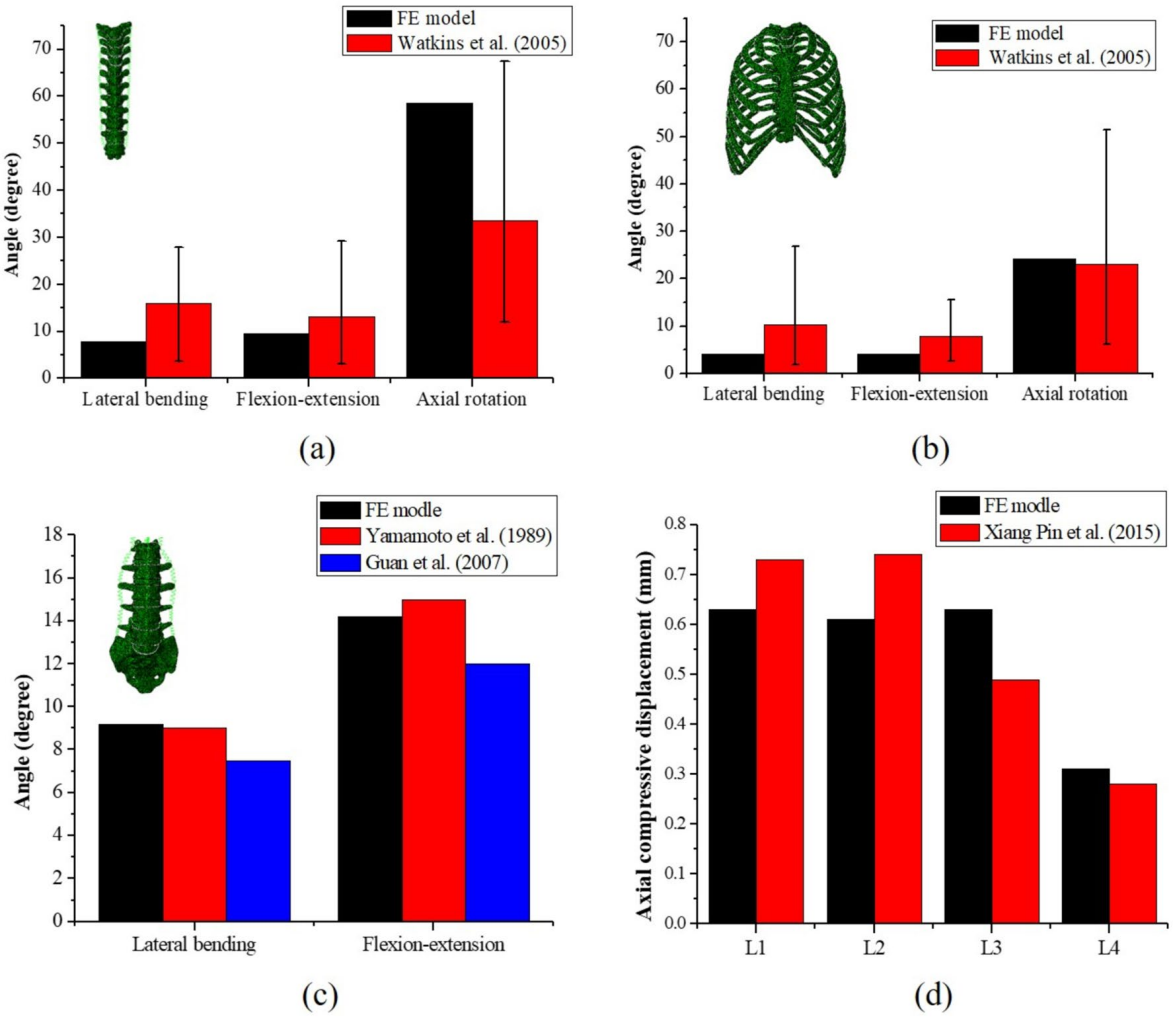
Validation of spinal FE models. (**a**) Thoracic spinal without rib cage rotational angle in lateral bending, flexion–extension, and axial rotation; (**b**) Thoracic spinal with rib cage rotational angle in lateral bending, flexion–extension, and axial rotation; (**c**) Lumbosacral vertebral (L1–S) rotational angle in lateral bending and flexion–extension; (**d**) The axial compressive displacement of L1–L4.

Validation was performed by comparison with published literature^11,25–27^ as shown in Fig. 2. It is difficult to verify the whole spinal FE model compared with cadaver experiments due to limited resources and research. Additionally, with different deformity characteristics found in different scoliotic spines, and there have been few studies on the thoracic spine in scoliosis, so validating scoliotic spine models is a unique challenge. Therefore, in this study, the FE models of thoracic and lumbar spines were verified against healthy spines. The FE models of thoracic spine (T1–T12) with and without a rib cage were compared to a cadaver experiment. The range of motions was measured at lateral bending with pure 2 Nm moment, flexion–extension with pure 2 Nm moment, and axial rotation with 5 Nm and 50 N preload. The results of the validation were strongly consistent with the cadaver experiment^11^. For the FE model of the lumbosacral spine (L1–S), lateral bending and flexion–extension with pure 4 Nm moment and axial compression with 400 N were used in this study. The lateral bending and flexion–extension results were similar to the cadaver experiments^25,26^. The axial compressive results of L1–L4 were similar to the finite element analysis by Xiang^27^. These results indicated that the modeling method used in this study is viable, and effective for establishing spinal FE models. The FE models of scoliosis with the same modeling method was therefore considered valid as well.

## Results

### Static analysis

**Figure 3:**
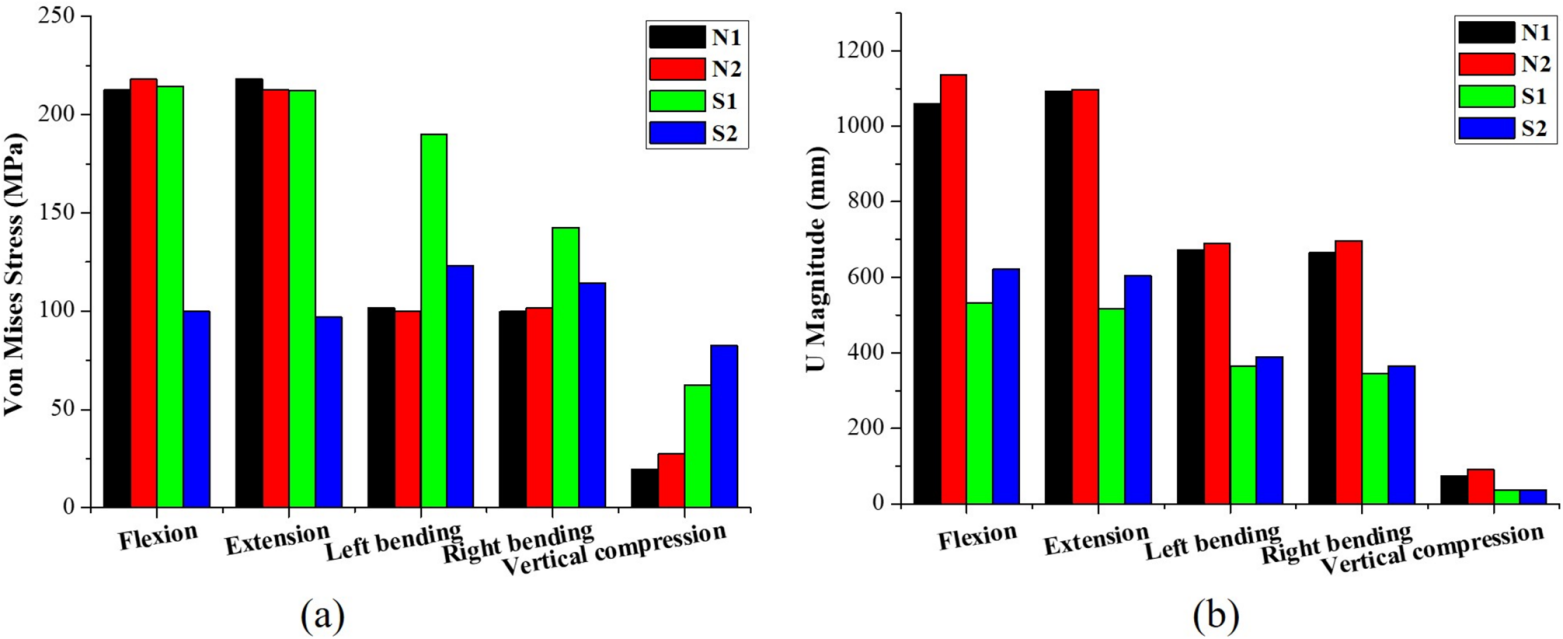
FE models subjected to five typical loadings. (**a**) Von Mises stress; (**b**) U Magnitude.

The results of the static analysis were shown in Fig. 3. The maximum values of VMS and U magnitude were used to analyze the mechanical responses of four models under five kinds of daily load. The VMS of N2 had a slight fluctuation in the four bending loadings, and a significant increase of approximately 38.5% for vertical compression compared to N1. The VMS of S2 had a strong decrease of approximately − 53.4% for flexion, − 54.2% for extension, − 35.1% for left bending and − 19.7% for right bending compared to S1. However, there was an increase in VMS of approximately 31.5% of S2 for axial compression compared to S1. The U magnitude of N2 increased slightly for four bending loadings and axial compressive loading, at 7.4%, 0.5%, 2.4%, 4.7% and 23.2% for flexion, extension, left bending, right bending and axial compression compared to N1, respectively. There was a significantly decrease in U magnitude for S2 by approximately 16.8% in flexion, 16.9% in extension, 6.8% in left bending, 5.9% in right bending and 2.1% in vertical compression compared to S1.

### Modal analysis results

The resonant frequencies and modes of modal analysis were shown in Table 2. Resonant frequencies affected vibrations in the axial direction of around 3–8 Hz. Therefore, the 4th order mode of N1, 3rd order mode of N2, 3rd order mode of S1 and 3rd mode of S2 were selected in this study. The resonant frequency of N2 was lower (− 0.27%) than N1. Similarly, the resonant frequency of S2 was lower (− 28.16%) than S1. The modes for N1 and N2 were mainly bending deformation, but those of S1 and S2 were axial stretching and twisting, respectively.

**Table 2:** Resonant frequencies and modes of N1, N2, S1 and S2, of around 3–8 Hz.

FE models	Order resonant	Frequency (Hz)	Description of modes of vibration of spine
N1	4	5.5383	Bending in the anterior–posterior direction at T8, T9 and T10
N2	3	5.5230	Bending in the anterior–posterior direction at T5–T12
S1	3	4.6203	Overall axial stretching
S2	3	3.7938	Twisting of the entire rib cage and spine in the axial direction

**Figure 4:**
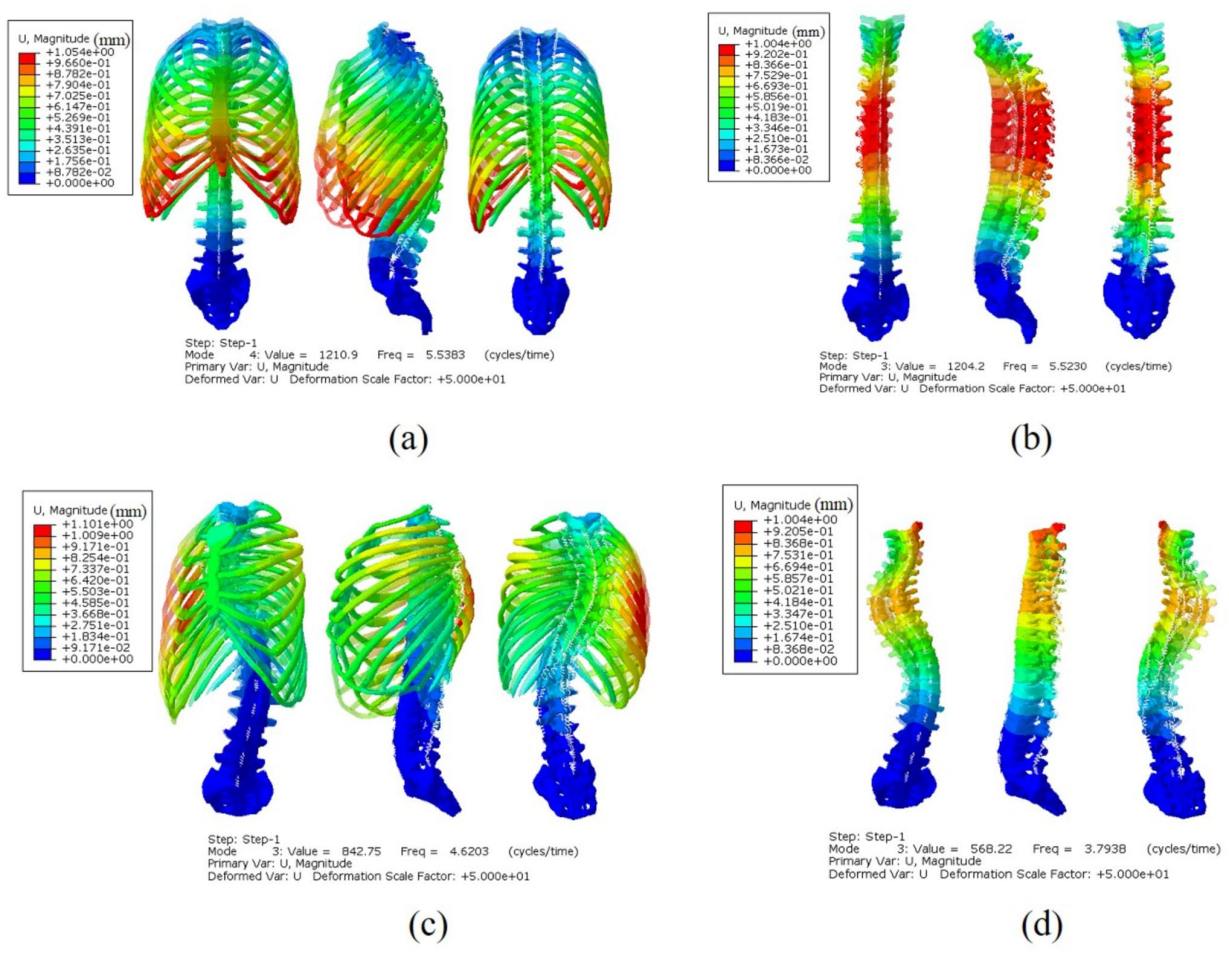
Displacement nephograms of modal shapes of the four FE models in the range 3–8 Hz. (**a**) 4th modal shape of N1; (**b**) 4th modal shape of N2; (**c**) 4th modal shape of S1; (**d**) 4th modal shape of S2.

The displacement nephograms of the modal shapes of N1, N2, S1, and S2 at approximately 3–8 Hz were shown in Fig. 4. The vertebrae and rib cage of the modal shapes were basically bilaterally symmetric with and without a rib cage in the normal spine. The modal shape of the rib cage was asymmetrical on the left and right sides in the scoliotic spine. The positions of the maximum amplitude of vibration of the four FE models were completely different. The maximum amplitude of vibration of N1 was mostly apparent on the outermost side of the 9th and 10th ribs, with an amplitude transition on the whole spine that was relatively stable. In N2 it was mostly concentrated on the T4–T6 segment, with an amplitude transition on the whole spine that was significantly different. In S1, it occurred at the outermost end of the right rib, with an amplitude transition of the spine that was relatively stable, and in S2 was mainly expressed in the T4–T6 convex side, with an amplitude significantly increased compared to the rest of segments.

### Steady-state analysis results

According to the modal analysis results, 3–8 Hz was selected as the frequency band for steady-state analysis. The scoliotic segment was the weak link in the spine, and demonstrated to be more susceptible to external vibration and damage. Therefore, representative nodes on the thoracic convex segment, T4–T6 of the lateral convex FE models, were selected as the principal research objects. The uppermost nodes of T4, T5, and T6 were selected as being representative for studying the vibration responses of the four FE models. The maximum amplitudes of the X-axis (coronal) direction, Y-axis (sagittal) direction, and Z-axis (axial) direction were selected for quantitative evaluation of the response to vibration of the convex section under external loading excitation.

**Figure 5:**
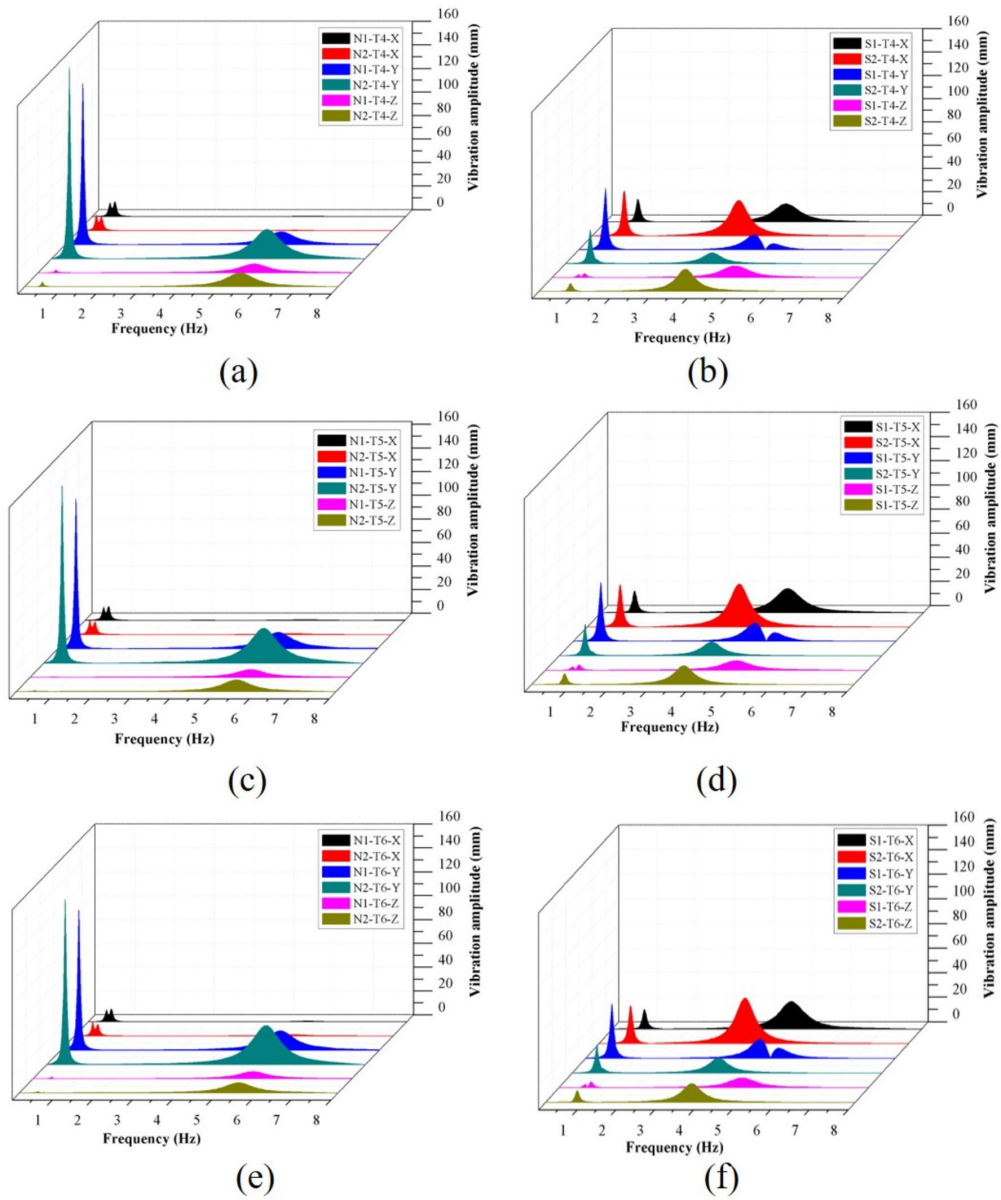
Frequency and displacement response values of the representative nodes T4, T5 and T6 in the X, Y and Z directions in the four FE models. (**a**) The response of T4 for N1 and N2; (**b**) The response of T4 for S1 and S2; (**c**) The response of T5 for N1 and N2; (**d**) The response of T5 for S1 and S2; (**e**) The response of T6 for N1 and N2; (**f**) The response of T6 for S1 and S2.

As shown in Fig. 5, representative nodes for each FE model presented clear vibration peaks over the 3–8 Hz range. The frequencies of the vibration peaks were consistent with the resonant frequencies observed in the respective modal analyses. For normal spine at all representative nodes, the maximum amplitudes of vibration at the resonant frequencies for N2-T6 were 73.85%, 100.24%, and 43.36% larger than N1-T6 in both X, Y, and Z directions, respectively. The maximum amplitudes of vibration at resonant frequencies of N2-T5 were 59.30%, 113.13%, and 51.26% larger than N1-T5 in both X, Y, Z directions, respectively. Similarly, the maximum amplitudes of vibration at the resonant frequencies of N2-T4 were 23.61%, 127.68% and 58.68% larger than N1-T4 in both X, Y, and Z directions, respectively. For the scoliotic spine at all representative nodes, maximum amplitudes of vibration of S2-T6 were 66.91%, 1.06% and 107.34% larger than S1-T6 in both X, Y, and Z directions, respectively. The maximum amplitudes of vibration for S2-T5 were 78.67%, − 10.23% and 101.24% larger than S1-T5 in both X, Y, and Z directions, respectively. Similarly, the peak amplitudes of vibration for S2-T4 were 94.75%, − 28.28% and 96.17% larger than S1-T4 in both X, Y, and Z directions, respectively. Compared N1 to S1, the peak amplitudes of vibration for S1 were larger than N1 in both directions and all representative nodes. Similarly, maximum vibrational amplitude of S2 was larger than N2 in both directions and representative nodes. In addition, the amplitude of vibration for T6 was the largest of T4–T6, followed by T5, with T4 the smallest.

## Discussion

Although the thoracic cage is an important component of the human skeleton, few studies have investigated its function in the human spine under dynamic loading. In addition, scoliosis is among the most harmful of spinal diseases. In this study, four FE models including the normal spine with and without a rib cage and a scoliotic spine with and without a rib cage were created. Static analysis was conducted to study static response to movements typically experienced in daily activities, and modal and steady-state analyses were performed to study the dynamic response to a vibration environment. The effect of the rib cage was then analyzed through comparison of the differences in static and dynamic characteristics of the four FE models.

In the validation of our FE model of a 27-year-old subject, the absence of a rib cage led to an increase rotation degree of 140% in axial rotation compared to a model with a rib cage. A previous experimental study using subjects with an average age of 56 years showed that in the absence of a rib cage there was a 130% increase rotation degree in axial rotation for the thorax compared to a thorax with a rib cage^28^, and its axial rotation behaviors, both with and without a rib cage, were similar to those of our model. However, in the referenced experimental data in our study, elderly subjects with an average age of 72 years were used to assess the axial rotation, and an increase of 31.4% in axial rotation was seen in the thorax without a rib cage^11^. To the best of our knowledge, it is unknown whether the rib cage provides a different amount of stability depending on age. By combining our data with previous study’s findings on axial rotation with aging, we found that there might be a correlation between age and rotation of motion. Further study will be needed to fully investigate the relationship between rotation of motion and age.

In static analysis, N1 and N2 demonstrated a small fluctuation in VMS and U magnitude. However, the VMS of S2 demonstrated a significant decrease compared with S1 for all daily loadings except for axial compression. For U magnitude, the deformation of N2 slightly increased compared to N1 under all static loadings. In addition, S2 experienced a great deal of deformation compared with S1 under all static loadings. This result implies that healthy and scoliotic spines without the rib cage exhibited reduced stability. Furthermore, this also indicates that a rib cage may increase the stiffness of a scoliotic spine^16,29^, increasing its stability^11,29^. At the same time, S2 experienced more deformation and smaller VMS than S1, and showed lower stiffness than S1, demonstrating that it was less able to resist deformation than S1. Moreover, the lower stiffness means that a smaller force is needed to produce a larger deformation than the structure with higher stiffness, according to concept of stiffness. In addition, this study indicated that a healthy spine with or without a rib cage experienced a slight fluctuation in VMS and increased U magnitude compared with a scoliotic spine. This implies that a healthy spine might be more stable than a scoliotic spine, which was consistent with other studies^14,30^. Overall, this study suggests that a rib cage plays an important role in maintaining spine stability under a series of static loadings, in particular, in the scoliotic spine.

The resonant frequencies of the four FE models were calculated using modal analysis. In the vibration frequency band of 3–8 Hz, the resonant frequency of N2 was lower (− 0.27%) than N1 model. Similarly, the resonant frequency of S2 was lower (− 28.16%) than S1. This result implies that N2 and S2 were more sensitive to vibration than N1 and S1, and that the rib cage could function to reduce the sensitivity of the spine to vibration. Moreover, the resonant frequencies of N1 and N2 were higher than S1 and S2 respectively. This result implies that a scoliotic spine is more sensitive to vibration than a normal spine, which is consistent with previous studies^4,30^. The observed modal shapes of N1 and N2 were mostly bending deformation, but modal shapes for S1 and S2 were axial stretching and twisting, respectively. These results demonstrated that the modal shape of the normal spine was completely symmetrical, but there was a large curvature to the right side of T6 which led to asymmetry of the modal shape in the X-axis. The rib cage’s role in spinal stability and stiffness has been proven by cadaver experiments which studied the range of motions (ROMs) of the spine with and without a rib cage under clinical rotation loads instead of dynamic loads^28,31^. In this study, our results also showed that both normal and scoliotic spines with a rib cage were more stable than spines without a rib cage in the vibration load. Although different loads were applied in this study and other previous studies, similar results among these studies were observed. In addition, the main influencing factor of modal shape was the spine’s structural characteristics and material properties. Andriacchi^9^ found that the rib cage increased the stiffness of the thoracic spine in all loading directions using a simplified computer simulation. Stiffness is related to stability, therefore, this study indicates that the scoliotic spine both with and without a rib cage had worse stability than a normal spine.

A number of studies have found that the rib cage contributes to the stability of a healthy spine^11,29^. In present study, the rib cage was also found to increase stability of the healthy spine, and even the scoliotic spine, under both static and vibration loadings. Furthermore, the rib cage in scoliosis provided a greater contribution to the scoliotic spine, with the vibration response between N1 and N2, and S1 and S2 all exhibiting different trends in vibration deformation along the X-, Y-, and Z-axes in steady-state analysis. In this study, maximum vibration amplitudes at N2 exhibited a significant increase in all three directions compared with N1. However, maximum vibration amplitudes in S2 presented a significant increase only in the X and Z directions compared with S1. These results imply that both normal and scoliotic spines are more unstable without a rib cage than those with a rib cage. Moreover, for a normal spine, the rib cage serves as an auxiliary support that can reduce the amplitude of the thoracolumbar spine in all three directions and weaken the sensitivity of the spine to external excitation, and improves the stability of the whole spine. However, in the scoliotic spine, the rib cage can significantly reduce amplitude in the X-axis and Z-axis, and greatly enhance stability in these directions. This indicates that the rib cage mainly contributes to stability in the X-axis and Z-axis for a scoliotic spine at resonant frequencies. These results suggest that the scoliotic spine exhibits a stronger tendency to vibrate in the X-axis and Z-axis due to the influence of the scoliotic segment than in a normal spine.

There are several limitations to this study. Firstly, only a limited number of subjects were used. Although the cohort size was small, we were still able to detect great differences between normal and scoliotic spines. We will continue to recruit more volunteers to confirm these initial observations in future studies and analyze the effects of the rib cage on stability for normal and scoliotic spines. Secondly, our study only focused on the vibration characteristics of normal and scoliotic spines, and did not conduct ROMs. ROMs analysis will be included to study the ROMs of whole spine for normal and scoliotic spines. In addition, only Lenke 1BN subjects were selected in this study, and other types should be examined to systematically compare the dynamic characteristics of the scoliotic spine. This will provide more valuable reference data for the study of the trend in stability of scoliotic spines and provide a resource for selection of protection from scoliosis. Another limitation is the scoliotic spine validation. It was difficult to verify the scoliotic spine validation due to a lack of experimental data and the range of deformations found in scoliotic spines. In future studies, scoliotic spines and experimental data of the same type should be compared to verify model validation. Although this study lacked validation of the scoliotic spine, the validation results for the normal spine strongly agreed with previous experimental data. Since the modeling method used was the same for normal and scoliotic spines in our study, there are good reasons to believe that the FE model of scoliotic spine was valid.

## Conclusions

This study demonstrated the effects of the rib cage on the static and dynamic responses of the typical Lenke 1BN scoliotic spine. The results implied that the rib cage was crucially important for maintaining stability in both normal and scoliotic spines during static and vibrational loads. The results indicated that scoliotic spines were more sensitive to external force, including static and dynamic vibration loads. Scoliosis patients should therefore avoid substantial spinal movement and long-term exposure to a vibration environment. These measures will help them reduce injury in daily life. This study demonstrates the importance of inclusion of the rib cage in experimental and computational models, and also provides significant insight from vibration analysis of the thoracic spine.

## Data Availability

Data can be obtained from the corresponding author by request.
